# Constraints on the anisotropic contributions to velocity discontinuities at ∼60 km depth beneath the Pacific

**DOI:** 10.1002/2017GC006850

**Published:** 2017-08-04

**Authors:** Catherine A. Rychert, Nicholas Harmon

**Affiliations:** ^1^ University of Southampton Waterfront Campus, Southampton UK

**Keywords:** surface waves, receiver functions, seismology, plate tectonics, Lithosphere Asthenosphere Boundary

## Abstract

Strong, sharp, negative seismic discontinuities, velocity decreases with depth, are observed beneath the Pacific seafloor at ∼60 km depth. It has been suggested that these are caused by an increase in radial anisotropy with depth, which occurs in global surface wave models. Here we test this hypothesis in two ways. We evaluate whether an increase in surface wave radial anisotropy with depth is robust with synthetic resolution tests. We do this by fitting an example surface wave data set near the East Pacific Rise. We also estimate the apparent isotropic seismic velocity discontinuities that could be caused by changes in radial anisotropy in S‐to‐P and P‐to‐S receiver functions and SS precursors using synthetic seismograms. We test one model where radial anisotropy is caused by olivine alignment and one model where it is caused by compositional layering. The result of our surface wave inversion suggests strong shallow azimuthal anisotropy beneath 0–10 Ma seafloor, which would also have a radial anisotropy signature. An increase in radial anisotropy with depth at 60 km depth is not well‐resolved in surface wave models, and could be artificially observed. Shallow isotropy underlain by strong radial anisotropy could explain moderate apparent velocity drops (<6%) in SS precursor imaging, but not receiver functions. The effect is diminished if strong anisotropy also exists at 0–60 km depth as suggested by surface waves. Overall, an increase in radial anisotropy with depth may not exist at 60 km beneath the oceans and does not explain the scattered wave observations.

## Key Points

- Strong shallow azimuthal anisotropy is required for 0–10 Ma seafloor, which would also give a strong shallow radial anisotropy signature
- An increase in radial anisotropy from 0 to 100 km depth beneath the oceans could be an artificial feature of surface wave models
- Negative velocity discontinuities imaged by scattered waves cannot simply be explained by an increase in radial anisotropy with depth

## 1 Introduction

The relatively short and simple history of the ocean lithosphere makes it the ideal place to study the plate. The oceanic lithosphere cools and subsides as it ages and moves away from the mid‐ocean ridge. Indeed, first‐order observations such as bathymetry and inverse heat flow increase as expected for a cooling plate, i.e., according to the square root of age [*Turcotte and Oxburgh*, 1967] at least beneath seafloor <70 My old. Older seafloor ceases to subside according to this model, although the exact cause and implications for the definition of the plate are debated [*Parsons and Sclater*, 1977; *Watts*, 1978; *Stein and Stein*, 1992].

Seismic imaging provides additional valuable constraints on plate thickness and structure at depth. Surface wave and body wave tomography image increasing seismic velocities in the lithosphere as it ages, and also increasing thickness of the seismically fast lithosphere, in general agreement with a thermally defined plate [*Ritzwoller et al*., 2004; *Priestley and McKenzie*, 2013; *Auer et al*., 2015]. In contrast, reflected and converted seismic phases image a strong, sharp discontinuity, typically ≥6% drops in seismic velocity with depth over <30 km depth, beneath large swaths of the Pacific [*Gaherty et al*., 1996; *Tan and Helmberger*, 2007; *Bagley and Revenaugh*, 2008; *Kawakatsu et al*., 2009; *Kumar and Kawakatsu*, 2011; *Rychert and Shearer*, 2011; *Tharimena et al*., 2017]. Although there is some spread in the scattered wave data, taken together no clear age‐depth trend is obvious, particularly beneath old seafloor [*Rychert et al*., 2012; *Tharimena et al*., 2017]. The majority of reported depths are centered around 60 km, shallower than predictions for the base of a thermally defined plate beneath old seafloor [*Rychert et al*., 2012; *Tharimena et al*., 2017]. The sharpness of the discontinuities suggested by many of these studies has been used to argue for melt or hydration in the mantle [*Kawakatsu et al*., 2009; *Rychert and Shearer*, 2009; *Schmerr*, 2012], which also affect the viscosity of the mantle [*Hirth and Kohlstedt*, 1995; *Hirth and Kohlstedt*, 1996; *Jackson et al*., 2006; *Faul and Jackson*, 2007], potentially allowing convection, and defining the tectonic plate [*Rychert et al*., 2005].

However, whether or not water or melt exist in the mantle at large enough volumes and whether or not they could explain the discontinuities is debated [*Faul*, 1997; *Karato and Jung*, 1998; *Behn et al*., 2009; *Hirschmann*, 2010]. Mechanisms that would affect seismic velocity without necessarily affecting viscosity have been proposed such as elastically accommodated grain boundary sliding [*Karato et al*., 2015] and the enhanced effects of near solidus conditions on seismic velocity [*Yamauchi and Takei*, 2016], although these are also debated. However, much attention has recently been paid to anisotropy. A change in anisotropy with depth could be frozen‐in, meaning the discontinuity observations may not necessarily correspond to the base of the plate. The coincidence in depth of an intermittent SS precursor discontinuity beneath the Pacific [*Schmerr*, 2012] with a peak in the gradient of the fast axis direction of azimuthal anisotropy from global surface waves was used to argue for an anisotropic origin to the discontinuities [*Beghein et al*., 2014]. Variations in SS precursor polarity with azimuth are expected for azimuthal anisotropy [*Rychert et al*., 2012, 2014]. However, discontinuity observations are overwhelmingly negative polarity, corresponding to velocity decreases with depth [*Gaherty et al*., 1996; *Tan and Helmberger*, 2007; *Bagley and Revenaugh*, 2008; *Kawakatsu et al*., 2009; *Kumar and Kawakatsu*, 2011; *Rychert and Shearer*, 2011; *Rychert et al*., 2012; *Tharimena et al*., 2017]. Therefore, it has also been suggested that the seismic discontinuities imaged by scattered phases near ∼60 km depth represent an increase in radial anisotropy with depth [*Burgos et al*., 2014; *Auer et al*., 2015], for which no variability in SS precursor polarity with azimuth is predicted [*Rychert et al*., 2012, 2014]. This notion has been further tested in the lab. Torsion experiments on olivine aggregates both with and without melt were combined with flow modeling to argue that the presence of melt beneath the ridge would inhibit shallow olivine fabric development [*Hansen et al*., 2016]. This would cause sharp increase in radial anisotropy with depth at 60 km in the oceanic lithosphere [*Hansen et al*., 2016].

The robustness of an increase in radial anisotropy with depth centered at 60 km is debatable. An increase in radial anisotropy from 0 to 100 or 150 km depth is suggested by global surface wave models [*Nettles and Dziewonski*, 2008; *Beghein et al*., 2014; *Burgos et al*., 2014]. However, regional studies support a different anisotropy depth structure. For instance, refraction experiments suggest 8% *P* wave anisotropy at shallow depth, just beneath the Moho at 7 km depth [*Raitt* et al., 1969; *Kodaira et al*., 2014]. Regional surface waves find azimuthal anisotropy decreasing with depth from 6 km down to 120 km beneath 70 My old lithosphere [*Lin et al*., 2016]. This azimuthal anisotropy would likely realize a radial signature in surface waves [*Montagner and Nataf*, 1986]. Whether an additional increase in anisotropy from 60 to 100 km depth exists beyond the high anisotropy magnitudes reported at 0–60 km depth is central to the debate.

Here we explore whether an increase in radial anisotropy alone can explain the observed discontinuities near 60 km depth. We do this in two ways. We first investigate whether an increase in radial anisotropy with depth centered at 60 km is a well‐resolved feature of surface wave models. We also test whether an increase in radial anisotropy with depth can explain the magnitude of the velocity contrasts reported by scattered wave imaging.

In situ Rayleigh wave azimuthal anisotropy inversions provide the best resolution of the depth distribution of anisotropy better than that typically attained for radial anisotropy. The reason for this is twofold. First, surface wave radial anisotropy is derived from the difference in apparent shear velocity structure between Love and Rayleigh waves. However, Rayleigh wave peak depth sensitivity increases with increasing period, while this is not necessarily true for Love waves. The inherently different sensitivity kernels of the waveforms adds uncertainty to the depth resolution of the shear velocity difference, making it nonunique. This problem is avoided with surface wave azimuthal anisotropy which is derived from variations in Rayleigh wave arrival times with azimuth, and thus only involving Rayleigh wave sensitivity kernels. In addition, in situ constraints can typically achieve higher resolution than global models, given closer station spacings. Azimuthal anisotropy caused by horizontal alignment of olivine, will produce a radial anisotropy signature, and so a well‐resolved azimuthal anisotropic structure can be translated to radial anisotropy under this assumption. In reality, a purely radial signature could also exist. However, our approach provides a minimum requirement for the magnitude of the radial anisotropy. This is particularly useful constraint for 0–60 km depth, since the hypothesized increase in radial anisotropy with depth would need to be in addition to the magnitude of the radial anisotropy at <60 km depth.

We start with the azimuthal constraints from a regional example on 0–10 My old lithosphere near the East Pacific Rise (EPR) [*Weeraratne et al*., 2007]. This is in part because the data set exists. Although it is also a reasonable choice, since the shallow anisotropy (0–60 km) observations at the ridge are expected to persist beneath older seafloor where the mantle remains relatively undeformed. We test the robust features of the azimuthal results with a series of inversions and forward modeling. We then calculate expected radial anisotropy signatures from the azimuthal anisotropy with depth. We then presume a radial anisotropy structure akin to our azimuthal anisotropy result and attempt to recover this radial anisotropic structure by inverting synthetic Rayleigh and Love wave dispersion data assuming a variety of start models.

**Figure 1:**
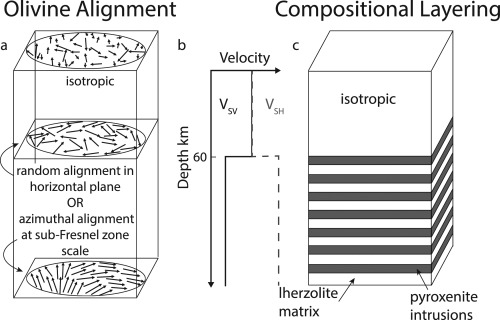
Cartoon illustrating two models that we tested to determine the maximum apparent velocity contrast in scattered wave imaging caused by an increase in radial anisotropy with depth. In both models, a 60 km thick isotropic layer is underlain by a an anisotropic layer in which *V_SH_* > *V_SV_*. (a) Radial anisotropy from olivine. In the shallow layer, olivine is randomly aligned, whereas in the deeper layer olivine is horizontally aligned at random azimuthal orientations (middle plate) or with azimuthal orientations that vary within the area of sensitivity of the wave, either sub bin size or sub‐Fresnel zone scale (bottom plate). The degree of alignment in the horizontal plane is indicated by the length of the arrows. (b) Schematic of the 1‐D shear velocity radial anisotropy structure that was tested. (c) Radial anisotropy from compositional layering. The shallow layer consists of an isotropic volume, whereas the deeper layer is composed of thin compositional layers. Lherzolite intruded by pyroxenite is an example of a very strong compositional velocity contrast difference, although we tried a range of intrusion velocities. A 50:50 compositional ratio is shown, although we also tested a range of melt intrusion‐to‐matrix ratios.

Finally, we use synthetic seismograms to predict the maximum apparent isotropic velocity contrast that would result from a radially anisotropic boundary that might be expected in the Earth. We test two models of radial anisotropy: (1) aligned olivine in the horizontal plane but with random azimuthal orientation at least within the area of sensitivity of the wave and (2) frozen‐in compositional layering (Figure 1). We focus on three phases (P‐to‐S and S‐to‐P conversions and SS precursors) frequently used to image upper mantle discontinuities.

## 2 Methods

### 2.1 Surface Wave Oceanic Azimuthal Anisotropy and its Associated Radial Signature

Azimuthal anisotropy of Rayleigh wave phase velocities was previously presented for the MELT [*Forsyth et al*., 1998] and GLIMPSE experiments [*Weeraratne et al*., 2007; *Harmon et al*., 2011] near 17°S on the East Pacific Rise from 0 to 10 Ma seafloor. Here we invert these results for shear wave azimuthal anisotropy with depth and relate these results to their predicted effective radial anisotropy after *Montagner and Nataf* [1986].

Azimuthal anisotropy for surface wave phase velocity can be described by

(1) $$X\left(k,\theta \right)=X_{1}\left(k\right)+X_{2}\left(k\right)\cos\left(2\theta \right)+X_{3}\left(k\right)\sin\left(2\theta \right)+X_{4}\left(k\right)\cos\left(4\theta \right)+X_{5}\left(k\right)sin(4\theta )$$

where *X* is a phase velocity as a function of wavenumber, *k*, and azimuth, *θ* [*Smith and Dahlen*, 1973] and *X*_1–5_ are the coefficients of the expansion. We only consider the isotropic and 2*θ* azimuthal variation (*X*_1–3_) as 4*θ* terms were previously found to be insignificant [*Weeraratne et al*., 2007].

The azimuthal variation in phase velocity can be related to the variation in elastic moduli with depth via equations (3) and (4) of *Montagner and Nataf* [1986]. We use a modified form neglecting 4*θ* terms:

(2) $$X\left(k,\theta \right)=C_{0R}(k)+\frac{1}{2C_{0R}}\left[R_{1}\left(k\right)+R_{2}(k)\cos\left(2\theta \right)+R_{3}\left(k\right)\sin\left(2\theta \right)\right]$$

where *C*_0R_ is the reference phase velocity and *R*_0–3_ are defined as follows:

(3) $$R_{0}(k)=\int_{0}^{\infty }\rho \left(z\right)\left({U\left(k,z\right)}^{2}+{V\left(k,z\right)}^{2}\right)dz$$

(4) $$R_{1}(k)=\frac{1}{R_{0}(k)}\int_{0}^{\infty }\left[dA{V(k,z)}^{2}+dC\frac{{U(k,z)\prime }^{2}}{k^{2}}+dF\frac{2U\left(k,z\right)\prime V(k,z)}{k}+dL{\left(\frac{V(k,z)\prime }{k}-U(k,z)\right)}^{2}\right]dz$$

(5) $$R_{2}\left(k\right)=\frac{1}{R_{0}(k)}\int_{0}^{\infty }\left[B_{c}\left(z\right){V(k,z)}^{2}+H_{c}\left(z\right)\frac{2U{\left(k,z\right)}^{\prime }V\left(k,z\right)}{k}+G_{c}(z){\left(\frac{V(k,z)\prime }{k}-U(k,z)\right)}^{2}\right]dz$$

(6) $$R_{3}\left(k\right)=\frac{1}{R_{0}(k)}\int_{0}^{\infty }\left[B_{s}\left(z\right){V(k,z)}^{2}+H_{s}\left(z\right)\frac{2U{\left(k,z\right)}^{\prime }V\left(k,z\right)}{k}+G_{s}(z){\left(\frac{V(k,z)\prime }{k}-U(k,z)\right)}^{2}\right]dz$$

where *U* and *V* are the radial and vertical displacement eigenfunctions of depth, *z*, of a fundamental mode Rayleigh wave at a given wavenumber, where ′ denotes vertical derivative. *A*, *C*, *F*, *L*, and *N* are the radially anisotropic parameters as defined by *Anderson* [1961] and *G_c,s_*, *H_c,s_*, and *B_c,s_* are the azimuthal anisotropy parameters defined by *Montagner and Nataf* [1986] in terms of the Chrisoffel Matrix in compact notation, *C_ij_*:

(7) $$A=\frac{3}{8}\left(C_{11}+C_{22}\right)+\frac{1}{4}C_{12}+\frac{1}{2}C_{66}$$

(8) $$C=C_{33}$$

(9) $$N=\;\frac{1}{8}\left(C_{11}+C_{22}\right)-\frac{1}{4}C_{12}+\frac{1}{2}C_{66}$$

(10) $$L=\frac{1}{2}\left(C_{44}+C_{55}\right)$$

(11) $$F=\frac{1}{2}\left(C_{13}+C_{23}\right)$$

(12) $$B_{c}=\frac{1}{2}\left(C_{11}-C_{22}\right)$$

(13) $$B_{s}=\left(C_{16}+C_{26}\right)$$

(14) $$G_{c}=\frac{1}{2}\left(C_{55}-C_{44}\right)$$

(15) $$G_{s}=C_{54}$$

(16) $$H_{c}=\frac{1}{2}\left(C_{13}-C_{23}\right)$$

(17) $$H_{s}=C_{36}$$

In addition, radial anisotropy may be related to horizontal and vertically polarized compressional (*V_PH_* and *V_PV_*, respectively) and shear velocity (*V_SH_* and *V_SV_*, respectively) and a fifth parameter η via the five elastic coefficients: *A*, *C*, *F*, *L*, and *N* defined by *Anderson* [1961]:

(18) $$V_{PH}=\sqrt{\frac{A}{\rho }}$$

(19) $$V_{PV}=\sqrt{\frac{C}{\rho }}$$

(20) $$V_{SH}=\sqrt{\frac{N}{\rho }}$$

(21) $$V_{SV}=\sqrt{\frac{L}{\rho }}$$

(22) $$\eta =\frac{F}{A-2L}$$

or recombined as:

(23) $$\xi =\frac{V_{SH}^{2}}{V_{SV}^{2}}=\frac{N}{L}$$

(24) $$\phi =\frac{V_{PV}^{2}}{V_{PH}^{2}}=\frac{C}{A}$$

We will use these definitions throughout the rest of the paper.

We solve for the azimuthal anisotropy as a function of depth in two steps using equations (1) and (2). For equation (1), we use the coefficients *X*_1–3_ from *Weeraratne et al*. [2007], and then we solve for the best fitting isotropic *V_SV_* structure, which minimizes the isotropic component (*X*_1_ − [*C*_0_ *_R_* + *R*_1_/(2*C*_0__*R*_)] = 0). Then we solve for the azimuthal anisotropy assuming a rotated coordinate system in the fast direction. The fast directions observed at all periods in the MELT and GLIMPSE study region were within error of the absolute plate motion of ∼100° azimuth. This assumption reduces equations (1) and (2) to a single cos(2[*θ*–100]) term. We define *X_c_* as the observed magnitude of the phase velocity azimuthal anisotropy (√[ $X_{2}^{2}$ + $X_{3}^{2}$]) in the fast direction, giving:

(25) $$X_{c}=\frac{R_{2}^{*}}{2C_{0R}}$$

*R*_2_* is used to denote the rotated coordinate system. We chose *C*_0__*R*_(*k*) as the average phase velocity in the region. We solve for *G_c_* but fix the ratio of the other derived elastic parameters *B_c_* and *H_c_* [*Montagner and Nataf*, 1986] based on ratios determined from a pure olivine crystal at each depth [*Abramson et al*., 1997].

For the isotropic inversion, we use partial derivatives and eigenfunctions calculated from DISPER80 [*Saito*, 1988] and a damped least‐squares approach [*Tarantola and Valette*, 1982] given by:

(26) $${\delta m}_{i+1}=\left(J^{T}C_{nn}^{-1}J+C_{mm}^{-1}\right)J^{T}C_{nn}^{-1}{\delta d}_{i}$$

where *δm_i_*_+1_ is the change in the model vector at iteration *i* + 1, *J* is the matrix of partial derivatives, *C_nn_* is the data covariance matrix that incorporates the formal error of the dispersion curve, *C_mm_* is the model covariance matrix, and *δd_i_* is the residual of the phase velocities at each period for Rayleigh waves. We assume a model covariance of 0.2 km/s and *V_p_*/*V_s_* is fixed at 1.8. We then use the best fit velocity structure to calculate the eigenfunctions and their derivatives required for modeling azimuthal anisotropy with depth.

**Figure 2:**
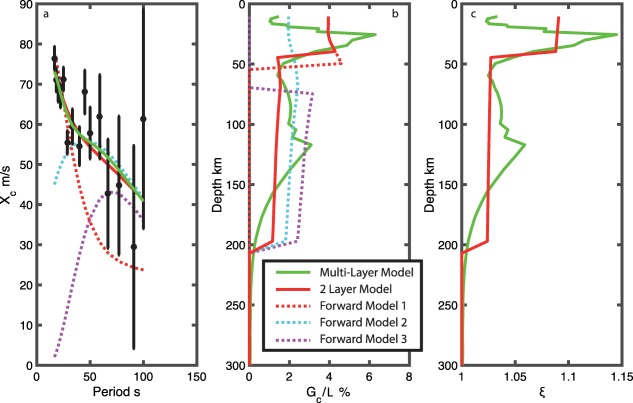
Depth distribution of azimuthal and radial anisotropy assuming olivine alignment from the MELT/GLIMPSE regions. (a) Amplitude of cos(2(*θ*‐100)) terms, from *Weeraratne et al*. [2007], with model predictions from multilayer inversion (green) and two‐layer model (red) and examples of alternate structure (dotted magenta, dotted red, and dotted cyan). (b) Value of *G_c_*/*L* in % as a function of depth from multilayer inversion (green), two‐layer inversion (red), and forward modeling of alternate structures (dotted). (c) Predicted *ξ* as a function of depth from multilayer inversion (green) and two‐layer inversion (red).

For the anisotropic inversion, we use a damped least‐squares inversion using equation (26) above, where *δm_i_*_+1_ and *δd_i_* are replaced with m the model vector and *d* the data vector, with no iteration to solve for the shear modulus difference, *G_c_* (equation (14)) as a function of depth. We invert for two different parameterizations: a multilayer model with the same number of layers as the shear velocity model and a two‐layer model. These parameterizations give a sense of the range of possibilities from standard inversion setup to a minimum structure estimate. We assume a model covariance of 10 GPa for *G_c_*, which allows for a relatively undamped solution. We also present predicted phase velocity amplitudes from forward models with anisotropy in only one of the layers (deep or shallow) to illustrate the misfit to the data, and highlight the robust features in the model (Figure 2, dashed lines).

We calculate the effective radial anisotropy from our azimuthal anisotropy results beneath the MELT and GLIMPSE study region assuming horizontally aligned olivine. We generate a Christoffel Matrix consistent with our models of *G_c_* by a weighted average between isotropic olivine (Voight average) and horizontally aligned olivine. We then convert the resulting Christoffel Matrix to its radial anisotropic equivalent using the expressions from *Montagner and Nataf* [1986], or here equations (7), (8), (9), (10), (11) and (23).

### 2.2 Surface Wave Radial Anisotropy Depth Resolution

We test the ability to resolve the form of radial anisotropy (shallow versus deep) given fundamental mode Love and Rayleigh wave depth resolution kernels. Specifically, we tried to recover a strong shallow radial anisotropy structure, like our inferred radial anisotropy structure above. To generate our synthetic model, we calculate the dispersion for the fundamental mode Love and Rayleigh waves from 16 to 200 s period from the best fit *V_SV_* model from the MELT and GLIMPSE study region, and a radial anisotropic structure with a Gaussian peak at 25 km depth of *ξ* = 1.10, extending down to 100 km depth, with no crustal anisotropy. We assume fixed relationships between the other anisotropic and isotropic parameters: *δ*ln*ϕ* = −1.5*δ*ln*ξ*, and *δ*ln*η* = −2.5*δ*ln*ξ*, relationships chosen from previous work [e.g., *Montagner and Anderson*, 1989; *Panning and Romanowicz*, 2006].

We invert the synthetic phase velocity dispersion for *V_SV_* and *ξ* using an iterative damped least‐squares approach [*Tarantola and Valette*, 1982] enforcing a smooth first derivative using equation (26), where *δd_i_* is the residual of the phase velocities at each period from 10 to 200 s, for Rayleigh and Love waves from the previous iteration. Partial derivatives are calculated using DISPER80 [*Saito*, 1988], and we assume a model covariance of 0.4 km/s for the *V_SV_* and 0.05 for *ξ*. We allow the model to iterate until the fit to the data is <0.01 km/s at all periods. Again we assume fixed relationships between the other anisotropic and isotropic parameters: *δ*ln*ϕ* = −1.5*δ*ln*ξ*, and *δ*ln*η* = −2.5*δ*ln*ξ*, relationships chosen from previous work [e.g., *Montagner and Anderson*, 1989; *Panning and Romanowicz*, 2006]. We do not allow anisotropy in the crust. We use three different starting models, one assuming there is no radial anisotropy, one assuming deep (100–200 km) radial anisotropy and shallow (<100 km) radial anisotropy.

### 2.3 Conversions/Reflections, Olivine a‐axes Randomly Aligned in the Horizontal Plane

We tested the maximum apparent velocity discontinuity that could result from a change in radial anisotropy caused by alignment of olivine a‐axes in the horizontal plane (Figure 1). In other words, we assume that the a‐axes are horizontal but have either random azimuthal distribution or an azimuthal distribution that is variable within the area of sensitivity (Fresnel zone or binned area) of the waveform being considered. Although, scattered waves are sensitive to impedance contrasts, i.e., changes in velocity and also density, we focus on velocity here, assuming constant density throughout our models. P‐to‐S and S‐to‐P conversions are relatively insensitive to density [*Rychert et al*., 2007], whereas reflections such as SS have sensitivity to both velocity and density [*Shearer and Flanagan*, 1999]. We compare the amplitude of the converted and reflected waveforms since it is the only waveform characteristic that varies in our models. The assumption of radial anisotropy with vertical symmetry axis, layering that is much thinner than the seismic wavelength, and also step‐function change from isotropy to anisotropy means there is no splitting or pulse width variability.

We considered a discontinuity between an isotropic layer at the surface and a deeper radially anisotropic layer to get the maximum effect from radial anisotropy alone. For the radially anisotropic layer, we assumed the olivine Christoffel Matrix coefficients corrected for pressure at 60 km depth [*Abramson et al*., 1997]. We averaged these coefficients according to *Montagner and Nataf* [1986]. We performed a weighted average with the Voigt average velocity, to explore the effect of varying the percentage of aligned olivine from 0% to 100%. For the isotropic layer, we use the Voigt average velocity of the radially anisotropic layer.

We calculated P‐to‐SV, SV‐to‐P, and SS precursor synthetic seismograms to consider reflections and conversions at the interface between the isotropic layer and a deeper radially anisotropic layer [*Keith and Crampin*, 1977]. The SH reflection coefficient may be calculated analytically at the boundary between the two layers and compared directly to isotropic equivalents. For the P‐to‐S and S‐to‐P conversions, the waves are initiated in a deeper half space with Voigt average velocity beneath the anisotropic layer, and the radial and vertical synthetic seismograms are rotated to P‐SV components using a free‐surface transfer matrix assuming the Voigt average velocity in the shallow layer [*Bostock*, 1998; *Cerveny*, 2001]. The amplitudes of the conversions are compared to rotated synthetic conversions created in the same way, except with the middle layer replaced by a range of isotropic velocities. We did this for a range of representative incidence angles.

### 2.4 Conversions/Reflections, Thin Compositional Layers

We also tested the maximum apparent velocity discontinuity that can result from radial anisotropy caused by compositional layering. In other words, alternating thin layers of rock compositions with fast and slow seismic velocities that are parallel to the Earth's surface, with vertical symmetry axis (Figure 1). To get effective radial anisotropy, the layers must be much thinner than the seismic wavelength. Then the resulting radially anisotropic Christoffel Matrix can be calculated using effective medium theory [*Backus*, 1962].

In our first test, we assume the thin layers exist in proportion to one another, a 50:50 ratio between the two compositions, since this gives the maximum apparent isotropic velocity contrast. We assumed a shallow lherzolite layer (*V_s_* = 4.78 km/s, *V_p_*/*V_s_* = 1.76) [*Hacker and Abers*, 2004] from 0 to 60 km depth in comparison to a deeper layer in which lherzolite is layered with another mineral of slower velocity. We vary *V_s_* of the second mineral and assume *V_p_*/*V_s_* = 1.8. We then use the Backus averaging to calculate the elastic coefficients in the radially anisotropic layer. We create synthetic seismograms for SS reflections, P‐to‐S conversions, and S‐to‐P conversions from the interface from the lherzolite layer and a deeper radially anisotropic layer. We determine the equivalent isotropic velocity contrasts analytically for SS and by comparison to rotated synthetic seismograms for P‐to‐S and S‐to‐P, in the same way as described above for the olivine case, although assuming a lherzolite velocity in the shallow layer. There is an inherent isotropic component to the velocity contrast between the lherzolite and the layered region that depends on the velocity of the second mineral in the layered region. To isolate the effect of the radial anisotropy, we calculate the Voigt average of the layered region and subtract the isotropic component of the velocity contrast between the lherzolite and the layered region from the total apparent contrast. The idea is that the isotropic layer would have the same velocity as the average velocity of the anisotropic layer if the observations of a discontinuity are caused purely by radial anisotropy.

We also present the case where there is a large velocity contrast between the individual layers, but the compositions are not necessarily at a 50:50 ratio. We assumed an example of a very strong compositional velocity contrast for the mantle, a lherzolite matrix (*V_s_* = 4.78 km/s, *V_p_*/*V_s_* = 1.76) and pyroxenite intrusion with a shear velocity that is 13.6% slower (*V_s_* = 4.13 km/s, *V_p_*/*V_s_* = 1.8) [*Hacker and Abers*, 2004]. We use Backus averaging [*Backus*, 1962] on the intruded region and calculate the apparent velocity contrast between the intruded region and a shallower layer of pure lherzolite for increasing pyroxenite‐to‐lherzolite ratios. SS contrasts are calculated analytically. P‐to‐S and S‐to‐P isotropic equivalents are calculated by comparison to isotropic examples, as described for the olivine case, but assuming lherzolite velocities in the shallow layer. Here again there is an inherent isotropic component to the velocity contrast that depends on lherzolite‐to‐pyroxenite ratio. To isolate the effect of the radial anisotropy, the Voight average of the layered region is calculated, and the isotropic component of the velocity contrast between the lherzolite and the layered region is subtracted from the total contrast.

## 3 Results

### 3.1 Surface Wave Azimuthal Anisotropy Model Results

We present our best fit azimuthal anisotropy as a function of depth from our two different parameterizations (Figures 2a and 2b). Both the multilayer and two‐layer damped least‐squares models fit the data equally well. We also show three forward model examples to illustrate how different features of the model affect the fit to the data, and also to highlight the features that are required by the data. Finally, we present the predicted radial anisotropy assuming olivine alignment in the mantle (Figure 2c).

The multilayer model has a strong peak in azimuthal anisotropy of up to 6% relative to the *V_SV_* model at 26 km depth, decreasing to 2–4% from 50 to 200 km depth, then rapidly decaying with depth. For the two‐layer model, we find a strong azimuthal anisotropy in a narrow region beneath the crust from 4% in the upper 40 km of the mantle, with 2% in the rest of the model down to 200 km (red solid line Figure 2). We experiment with different layer thicknesses, and find that the strength of anisotropy in the shallow layer trades off with layer thickness, i.e., a thinner shallow layer needs stronger anisotropy. However, models with a 30–50 km thick shallow layer give the best fit to the data. These models also have good visual agreement with the multilayer model.

Strong, shallow azimuthal anisotropy (≥4% at <50 km depth) is required in both the multilayer and two‐layer models to explain the high values of peak‐to‐peak anisotropy observed from 16 to 20 s period. The difference between the multilayer and two‐layer model highlight that the exact form of the azimuthal anisotropy in depth is not well‐resolved. However, testing with forward models also shows that certain parts of the model are robust. For instance, strong shallow anisotropy is required, since models with no or weak shallow anisotropy (0–3%) do not fit the data (magenta and cyan dotted lines, Figure 2). From 50 to 200 km depth, moderate anisotropy (∼2–3%) is needed to satisfy the peak‐to‐peak anisotropy from 22 to 100 s period in all the best fit models. Alternatively, with 4% anisotropy in the upper 50 km and 0% deeper, the predicted *X_c_* values rapidly go to zero at the longer periods and do not fit the data at >30 s period (red dotted line, Figure 2). Thus, at a minimum, two layers of anisotropy are required, with moderate anisotropy from 50 to 200 km and greater anisotropy from 0 to 50 km.

The predicted radial anisotropy (Figure 2c) for the multilayer model ranges from a peak ξ value of 1.14 at 25 km depth to the background value of 1.04, with the two‐layer model having a ξ value of 1.09 at its peak at maximum in the upper 15–50 km and a value of 1.03 down to 200 km.

### 3.2 Surface Wave Radial Anisotropy Results

Given our results from young oceanic lithosphere which imply greater azimuthal and radial anisotropy at shallow depths, we examine the ability to resolve such a structure. We use our best fit *V_SV_* structure for the East Pacific Rise and inserted a strong Gaussian shaped radial anisotropy with a peak of *ξ* = 1.10 in the upper 100 km into the model and calculated a synthetic dispersion curve. This synthetic test loosely approximates our best fit anisotropy with a simple and smooth structure. We attempt to recover this radial anisotropy from the dispersion curves using a standard least‐squares inversion scheme described above and a variety of starting models.

**Figure 3:**
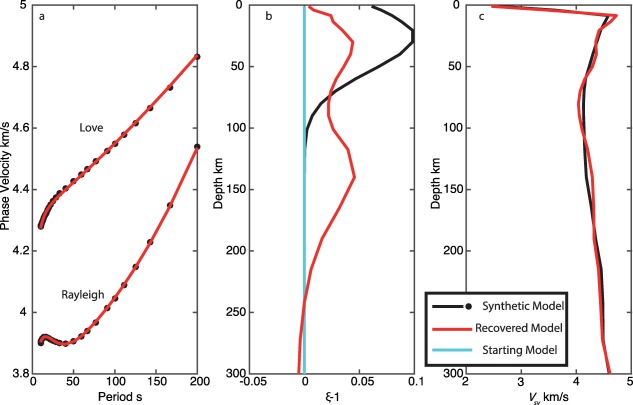
Recovery tests for shallow radial anisotropy: start model with zero radial anisotropy. (a) Love and Rayleigh wave dispersion, black indicates synthetic data, and red indicates best fit inversion model. (b) Synthetic model for *ξ* (black), starting model (cyan), and recovered best fit model (red). (c) Vertical shear velocity model for synthetic model (black) and best fit model (red).

In our first test, we assume no radial anisotropy and the best fit *V_SV_* model in our starting model. The inversion satisfactorily fit both the synthetic Love and Rayleigh dispersion curves. However, the model converges on a double peaked model for *ξ*, with peaks at 30 and 140 km depth with values of 1.05 and 1.04 with a trough at 80 km (Figure 3).

**Figure 4:**
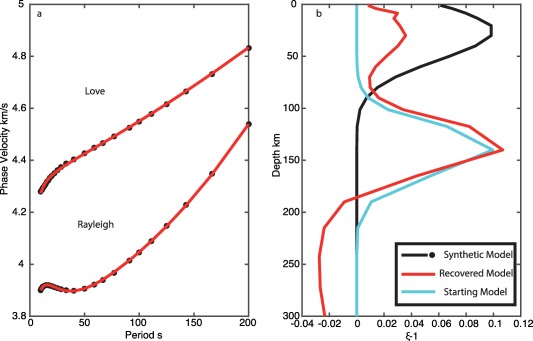
Recovery tests for shallow radial anisotropy: deep radial anisotropy start model. (a) Love and Rayleigh wave dispersion, black indicates synthetic data, and red indicates best fit inversion model. (b) Synthetic model for *ξ* (black), starting model (cyan), and recovered best fit model (red). Although not shown, the vertical shear velocity model agrees well with the synthetic, within error as in Figure 3.

In our second test, we use a starting model with deep Gaussian shaped radial anisotropy, peaking at *ξ* = 1.10 at 150 km depth and the best fit *V_SV_* model (Figure 4). The model also satisfactorily fits the synthetic dispersion curves; however, the model maintains the strong peak at 150 km depth. The inversion recovers only a minor peak at 30 km of up to 1.02, and weak radial anisotropy with *V_SV_* faster *ξ* = 0.98 at depth >200 km.

**Figure 5:**
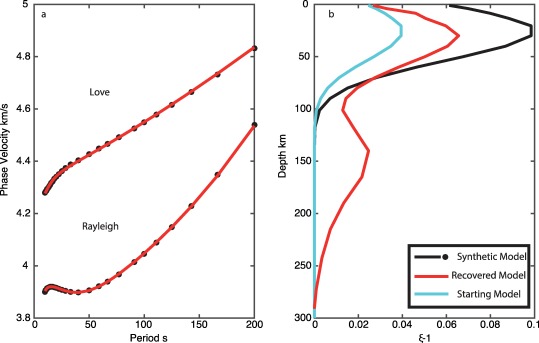
Recovery tests for shallow radial anisotropy: shallow radial anisotropy start model. (a) Love and Rayleigh wave dispersion, black indicates synthetic data, and red indicates best fit inversion model. (b) Synthetic model for *ξ* (black), starting model (cyan), and recovered best fit model (red). Although not shown, the vertical shear velocity model agrees well with the synthetic, within error as in Figure 3.

Finally, we assume a starting model with a radial anisotropy structure with shallow radial anisotropy, like the true model, but reduced in magnitude (*ξ* = 1.05) and the best fit *V_SV_* model (Figure 5). The inversion fits the data; although, a secondary peak in the data centered at 150 km depth is also generated in the model with *ξ* = 1.025, with a trough at 100 km depth.

**Figure 6:**
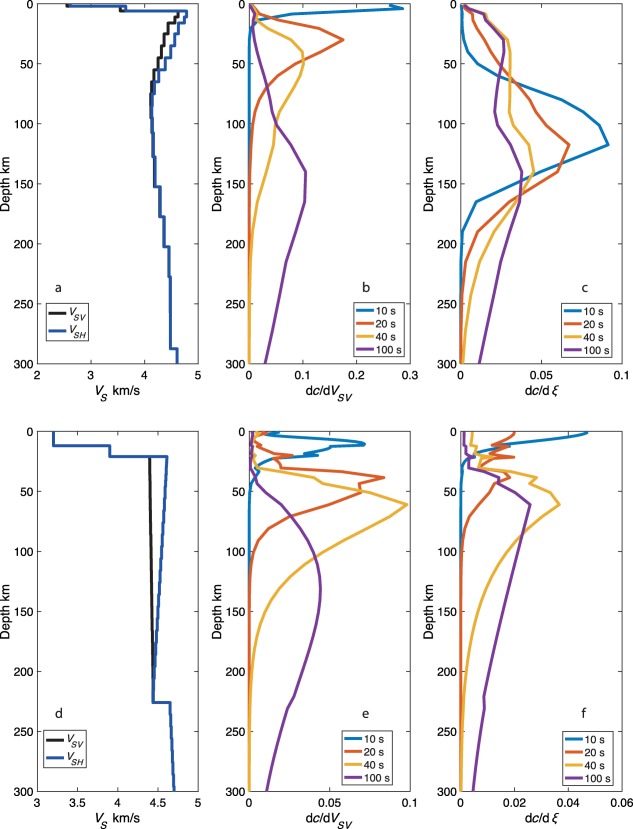
Comparison of *V_SV_* and *V_SH_* models and sensitivity kernels for *V_SV_* and *ξ* as a function of depth (a–c) for the GLIMPSE synthetic example and (d–f) for PREM for 10, 20, 40, and 100 s period [*Dziewonski and Anderson*, 1981].

We present the sensitivity kernels for *V_sv_* and *ξ* for the GLIMPSE best fit model as compared to PREM [*Dziewonski and Anderson*, 1981] to highlight the origin of the deeper peak in *ξ* (Figure 6). The *V_SV_* sensivities are similar between PREM and the GLIMPSE model, showing the expected increase in the depth in peak sensitivity with increasing period (Figures 6b and 6e). There is a marked difference in *ξ* sensitivity between PREM and the GLIMPSE synthetic example (Figures 6c and 6f). In the GLIMPSE synthetic example, the strongest sensitivity to *ξ* is at 10 s period and has a peak at 118 km depth, with the long periods also having peak sensitivity down to 150 km depth. At 40 and 100 s period, there is a second shallow peak in sensitivity at ∼30 km depth in the GLIMPSE model. PREM has shallower peak sensitivity at 20–100 s periods at 40–60 km depth, and at 10 s period peak sensitivity is in the crust. The greater sensitivity at 120–150 km depth to *ξ* in the GLIMPSE model arises from the strong low velocity zone in that model. This suggests that the existence of a low velocity zone in the best fit model could result in a deep peak in the radial anisotropy sensitivity kernel, and also the artificial deep radial anisotropy peak in our recovery tests above.

### 3.3 Conversion and Reflection Results

**Figure 7:**
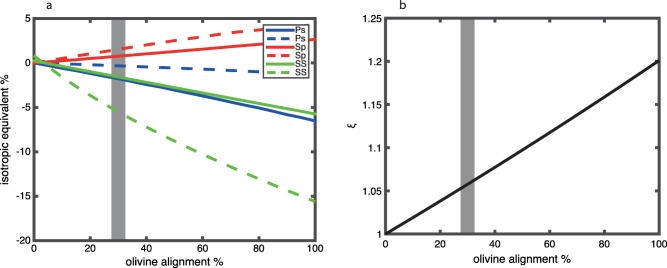
Predictions for a discontinuity from a shallow isotropic layer and a deeper radially anisotropic layer with olivine a‐axes randomly aligned in the horizontal plane. (a) Apparent isotropic velocity contrast from scattered wave imaging. The result is shown for P‐to‐S converted phases (blue), S‐to‐P converted phases (red), and SS bottom side reflections (green) for a typical range of incidence angles, small (solid) to large (dashed). (b) Equivalent *ξ* for the anisotropic layer. 30% aligned olivine is expected from natural sample analysis, as indicated by a grey line [*Mainprice et al*., 1998].

We present the apparent isotropic velocity contrasts from a radially anisotropic discontinuity in P‐to‐S, S‐to‐P, and SS imaging caused by olivine a‐axis alignment in the horizontal plane, with random azimuthal orientations (Figure 7). The apparent magnitude of the velocity contrast increases with increasing percentages of aligned olivine. We present results for a wide range of aligned olivine content (0–100%). However, the fabrics of natural olivine aggregates from fast spreading ridges and their estimated elastic coefficients suggest average anisotropic strengths corresponding to 6.5% anisotropy, i.e., fast versus slow shear velocity [*Mainprice et al*., 1998]. This corresponds to about 30% aligned olivine. Assuming this is representative of the Earth, this suggests olivine can explain apparent isotropic velocity contrast magnitudes smaller than 2% for P‐to‐S and S‐to‐P imaging and smaller than 5.5% for SS imaging (Figure 7). The apparent sign of the velocity discontinuity depends on the waveform. We find an apparent positive discontinuity (velocity increase with depth) for S‐to‐P and apparent negative discontinuities (velocity decrease with depth) for P‐to‐S and SS imaging. Converted S‐to‐P waveforms are predicted to be opposite polarity from P‐to‐S and SS at isotropic boundaries, i.e., positive at velocity decreases with depth, although S‐to‐P waveforms are often inverted for convention and comparison purposes to P‐to‐S. Here when we refer to a velocity contrast, we are referring to the discontinuity itself, with positive contrasts corresponding to velocity increases in depth, and vice versa.

**Figure 8:**
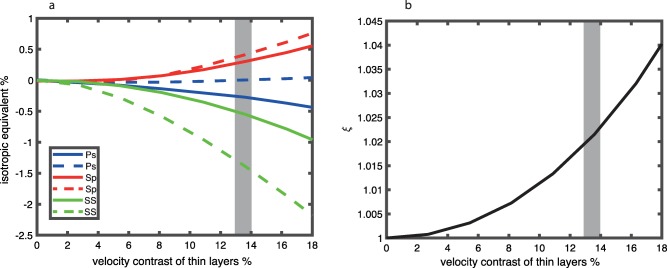
Predictions for a discontinuity from a shallow isotropic layer and a deeper radially anisotropic layer composed of thin compositional layers of alternating high and low velocity, fixed at equal compositional proportions (50:50). (a) Apparent isotropic velocity contrast from scattered wave imaging. The result is shown for P‐to‐S converted phases (blue), S‐to‐P converted phases (red), and SS bottom side reflections (green) for a range of incidence angles, small (solid) to large (dashed). The isotropic contrast between the layers has been removed to illuminate the effect of the radial anisotropy. Grey line indicates largest realistic velocity contrast from frozen‐in compositional layering given Earth's mineral assemblages, for pyroxenite in comparison to lherzolite [*Hacker and Abers*, 2004]. (b) Equivalent *ξ* for the anisotropic layer.

We present the apparent velocity contrast from a radially anisotropic discontinuity that results from compositional layering in which the layers exist in equal proportion to each other, 50:50 (Figure 8). Apparent isotropic discontinuity magnitude increases as the velocities of the layers are increasingly different. We show up to 18% velocity contrast between the compositional layers. However, the lherzolite‐pyroxenite contrast is one of the strongest velocity contrasts we can expect at these depth from composition alone, pyroxenite being 13.6% slower than lherzolite [*Hacker and Abers*, 2004]. Assuming the lherzolite‐pyroxenite velocity contrast between the layers and 50:50 proportion of the compositions, the apparent isotropic velocity contrast magnitude would be smaller than 0.5% in P‐to‐S and S‐to‐P imaging and smaller than 1.5% in SS imaging. Again, the apparent polarity of the discontinuity depends on the waveform being considered.

**Figure 9:**
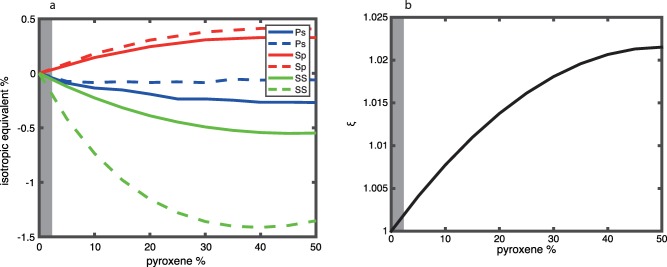
Predictions for a discontinuity from a shallow isotropic layer and a deeper radially anisotropic layer composed of thin compositional layers of alternating high (lherzolite) and low (pyroxenite) velocity at variable compositional proportions. (a) Apparent isotropic velocity contrast from scattered wave imaging. The isotropic contrast has been removed to illuminate the effect of the radial anisotropy. The proportion of pyroxenite may be quite small (<1%, grey line), depending on how much melt may be retained in the mantle without escaping to the surface. We show the effect on P‐to‐S (blue), S‐to‐P (red), and SS bottom side reflections (green) for a range of incidence angles, small (solid) to large (dashed). (b) Equivalent *ξ* for the same range of thin intruded pyroxenite.

We present the apparent velocity contrast from a radially anisotropic discontinuity that results from compositional layering in which the velocity of the layers is fixed as the lherzolite‐pyroxenite contrast [*Hacker and Abers*, 2004], but the proportion of one layer to the other varies (Figure 9). The apparent isotropic discontinuity increases as the proportion becomes more equal, i.e., as it approaches a 50:50 ratio. The realistic percentage of an intruded layer may be quite low, for instance, if melt fractions greater than 1%, escape to the surface [*Faul*, 1997]. In this case, the magnitude of the apparent velocity contrast from the radial anisotropy is very small, <0.1%, although other possible scenarios are discussed in greater detail in subsequent sections. Again, the apparent polarity of the discontinuity depends on the waveform being considered.

## 4 Discussion

### 4.1 Implications of Strong Shallow Azimuthal Anisotropy for Surface Wave Radial Anisotropy

Our inversion for strong shallow azimuthal anisotropy within the ocean lithosphere agrees with several other constraints from regional studies, which show this relatively consistently. Specifically, the strong shallow azimuthal anisotropy from the MELT and GLIMPSE regions is consistent with several other in situ regional results from surface waves [*Takeo et al*., 2013; *Lin et al*., 2016] and also active source constraints from just beneath the crust [*Raitt et al.*, 1969; *Kodaira et al*., 2014] from a range of ages. We have shown here that this type of strong azimuthal anisotropy would necessarily have a radial anisotropy signature if it results from the alignment of olivine in the horizontal plane. The radial anisotropy predicted from the azimuthal anisotropy assuming horizontally aligned olivine is significant, with *ξ* values up to 1.14 within the upper ∼50 km. The formation of strong shallow anisotropy at young seafloor ages is likely preserved in the lithosphere as the plate ages as implied by *Lin et al*. [2016] study on much older seafloor. This represents a minimum requirement for the degree of radial anisotropy at shallow depths (0–60 km) beneath the seafloor.

Such shallow azimuthal and radial anisotropy is opposite to many current global models [*Beghein et al*., 2014; *Burgos et al*., 2014; *Auer et al*., 2015], but consistent with models such as PREM [*Dziewonski and Anderson*, 1981]. Long periods used in global models (>30 s) cannot resolve shallow anisotropy signatures visible in the short period data (<22 s). Our synthetic tests suggest that if global models incorporated this strong shallow anisotropy in their models the strength of the global anisotropic anomaly at depth would be diminished owing to the overlapping depth sensitivities (e.g., Figures 3, 4, 5). Therefore, while we cannot preclude the possibility of a moderately strong deep (∼150 km) anisotropic layer, a large additional increase in anisotropy with depth over the already high magnitudes that our result and those of others [e.g., *Lin et al*., 2016] suggest at shallow depth (0–60 km) to explain the observed discontinuities is not likely. Finally, a model in which an isotropic layer is sandwiched between a strong shallow (sub‐Moho) and a strong deep (at >100 km depth) layer of anisotropy, to allow for the strong increase that would be required to explain the strength of the observed discontinuity at ∼60 km is not supported at least by the regional EPR result since moderate anisotropy, not isotropy, is required in the intermediate depth range (20–60 km).

Inverting synthetic data using a standard least‐squares inversion technique indicate that it is difficult to recover strong radial anisotropy at depths <100 km. An artificial deeper peak in radial anisotropy at ∼150 km depth developed regardless of the starting model. The deep peak in our results is spurious, and models fit the data equally well with or without it. The deeper peak in our result is deeper than that in some global models, but as we have demonstrated here the exact depth of the peaks in anisotropy is not well‐resolved and tied to the presence of a low velocity zone. This feature arises due to the depth sensitivity kernels of the fundamental mode surface waves used here, and would be difficult to eliminate in practice using least‐squares inversion. The strong sensitivity in the 100–200 km depth range for Love waves here arises from the presence of a strong low velocity zone, which acts as a waveguide for SH. We were only able to recover the true model, when we assumed a starting model very close to the true model. Combining constraints from azimuthal anisotropy and its strength with depth may help to illuminate how much radial anisotropy is required at shallow depths. To resolve the depth dependence requires short period observations that can only be made with in situ observations.

The strong shallow anisotropy of the regional EPR result is not predicted by viscous mantle flow models. For example, anisotropy is expected to be well developed near the mid‐ocean ridge (within 100 km) with the strongest anisotropy developing between 50 and 150 km depth [*Blackman and Kendall*, 2002] for both the active and passive flow ridge models. Strong shallow anisotropy in viscous flow models is suppressed by high viscosities due to the low temperatures in the upper 30–50 km of the model, which prevents significant shear strains and anisotropic alignment. Our strong shallow anisotropy similarly conflicts with a result that incorporates new lab experiments into an ocean lithosphere flow model, arguing that the fabric inhibiting effects of melt in the upper 60 km would reduce radial anisotropy [*Hansen et al*., 2016]. Varying the choices of activation energy and volume might enhance deformation at shallower depths [*Hedjazian et al*., 2017], although overall a better link between mantle rheology is required to reconcile the geodynamic predictions with the seismic observations.

One possible explanation for the shallow anisotropy is that strain could be localized over narrow regions such as a shear zone, which is not accounted for in simple mantle flow models. Strong azimuthal anisotropy in the western Pacific was interpreted as a shear zone at the base of the crust caused by strong active mantle upwelling near the Paleo spreading center [*Kodaira et al*., 2014]. A shear zone has also been hypothesized at the base of a 70 km thick lithosphere beneath the Hikurangi Plateau based on seismic discontinuities, suggesting sharp differential lithosphere‐asthenosphere motion on older seafloor [*Stern et al*., 2015]. The presence of these shear zones suggests there may be physics of the earth that is not encapsulated in viscous geodynamic models that needs to be incorporated to understand seismic anisotropy at a macro‐scale.

### 4.2 Apparent Isotropic Discontinuities in Conversions and Reflections From a Change in Anisotropy

Overall, the largest apparent velocity contrasts from an increase in radial anisotropy with depth are attained in the model with olivine alignment in the horizontal plane (Figure 7). The magnitudes of the predicted apparent velocity contrast from SS precursors (5.5% for the largest incidence angles) get close to the magnitudes reported by other SS studies which are typically ≥6% [*Rychert and Shearer*, 2011; *Schmerr*, 2012; *Tharimena et al*., 2017]. The magnitudes of the predicted apparent velocity contrasts from P‐to‐S and S‐to‐P conversions are still too small (<2%) to explain the 7–8% contrasts from observations [*Kawakatsu et al*., 2009].

In addition, the sign of the apparent contrast varies depending on the phase being considered. For instance, S‐to‐P observations of negative velocity contrasts [*Kawakatsu et al*., 2009; *Kumar and Kawakatsu*, 2011] could not be equated with those from SS bounces [*Rychert and Shearer*, 2011; *Schmerr*, 2012; *Tharimena et al*., 2017] or P‐to‐S conversions [*Kawakatsu et al*., 2009; *Rychert and Shearer*, 2009] if the observations are caused by only an increase in radial anisotropy with depth. The same phenomenon is true for layering, i.e., a different predicted apparent sign for S‐to‐P discontinuity in comparison to a discontinuity P‐to‐S and SS bounces. We also note that if we relax our velocity assumptions, i.e., those that correspond to the composition assumption of olivine, for example, a different *η* could be achieved. A different *η* can give a different sign discontinuity for a given converted or reflected phase, although typically this causes the other phases we investigated to also be polarity reversed. Overall, this suggests that if these observations of negative velocity contrasts from converted phases and SS bounces are taken as one, they cannot be due to an anisotropic variation alone.

It is not possible to produce apparent large velocity contrasts from frozen‐in compositional layering, even if the velocity contrast between the layers is very large and/or the proportion of the layers is equal (Figures 8 and 9). If an extreme velocity drop (13.6%) from a lherzolite matrix to a pyroxenite intrusion is assumed [*Hacker and Abers*, 2004] and intrusion to matrix ratios are small, reflecting small mantle melt fractions, <1% [*Faul*, 1997], then the apparent velocity contrast is very small <0.1%. Alternatively, greater melt percentages could accumulate at a depth of neutral buoyancy [*Sakamaki et al*., 2013] with larger melt percentages frozen‐in instantaneously. Greater melt intrusion‐to‐matrix ratios could also be achieved if melt intrusions are frozen‐in progressively over time. However, even if higher intrusion‐to matrix‐ratios can be realized, the maximum apparent velocity contrast for a radially anisotropic effect is still small, <0.5% for P‐to‐S and S‐to‐P imaging and <1.5% for SS imaging, for a 50:50 matrix‐to‐intrusion ratio. This is far too small to explain the 7–8% velocity contrasts observed using P‐to‐S and S‐to‐P receiver functions [*Kawakatsu et al*., 2009] as well as the typically 6% or greater velocity contrasts reported from the large Pacific transect studies [*Gaherty et al*., 1996; *Tan and Helmberger*, 2007] and SS precursors [*Rychert and Shearer*, 2011; *Schmerr*, 2012; *Tharimena et al*., 2017].

Of course, if instead of frozen‐in compositional layers, there is layered mantle melt, it would produce a larger contrast between the melt and the solid matrix, and a larger overall effect on radial anisotropy [*Kawakatsu et al*., 2009]. However, unless the melt also exists in the cold lithosphere from 0 to 60 km, which is not likely, this model would also include an increase in melt content from the shallow to the deep layer. The presence of melt would also significantly decrease the isotropic seismic velocity of the deeper layer, and this would be the dominant effect [*Hammond and Humphreys*, 2000]. In other words, this could no longer be considered a purely radially anisotropic discontinuity.

The effects of olivine alignment and compositional layering could both exist at depth. The effects may not necessarily be additive. For instance, >1% partial melt may inhibit olivine fabric development [*Hansen et al*., 2016]. Alternatively, if the intrusion‐to‐matrix ratio reaches 50:50 by the addition of intrusions over time with melt fractions not exceeding 1%, it would then add <0.5% to apparent P‐to‐S and S‐to‐P receiver function contrasts, and it would add <1.5% to apparent SS imaging contrasts that might exist from olivine alone. This type of progressive addition is speculative and needs further investigation through modeling.

Finally, we emphasize that based on our analysis of surface wave constraints on azimuthal anisotropy in the oceanic lithosphere, there is not likely zero or even small radial anisotropy at 0–60 km depth. Therefore, our predictions for apparent scattered phase contrasts from radial anisotropy assuming isotropy from 0 to 60 km are maxima that would likely be significantly diminished in the presence of the shallow anisotropy suggested by surface waves.

## 5 Conclusions

Strong azimuthal anisotropy at 0–60 km depth is required by in situ, regional surface wave results that would also realize a strong radial anisotropy (*ξ* = 1.09) if it is caused by horizontal olivine LPO. However, an additional large increase in radial anisotropy from 60 to 100 km is not supported by regional constraints. Synthetic seismogram modeling of P‐to‐S and S‐to‐P converted phases and SS precursor reflections suggests moderate apparent isotropic velocity contrasts (<6%) are predicted for the contrast between an isotropic layer and a deeper radially anisotropic layer where olivine a‐axes randomly aligned in the horizontal plane. Radial anisotropy from shape preferred orientation of frozen‐in compositional layering can only explain extremely small apparent velocity contrasts (<1.5%) for expected mineral assemblages in the Earth. However, these contrasts would be diminished if moderate anisotropy also exists at 0–60 km depth, as suggested by the surface wave constraints. The apparent polarity of a radially anisotropic discontinuity varies depending on the phase that is considered so that reports from receiver functions and SS precursors cannot be equated and explained by radial anisotropy. Taken together both surface and body wave testing indicate that a purely radially anisotropic boundary cannot simply explain the strong, sharp, and consistently negative velocity discontinuity near 60 km depth by reflected and converted body waves. Another mechanism is required.
